# Commentary: Management Strategies for POSEIDON Groups 3 and 4

**DOI:** 10.3389/fendo.2020.00034

**Published:** 2020-02-04

**Authors:** Robert Fischer, Vera Baukloh

**Affiliations:** Fertility Center Hamburg, Hamburg, Germany

**Keywords:** clinical management guidance, poor responder, individual stimulation, chromosomal problem, live birth rate

As elaborated in the excellent paper by Haahr et al. (1) the POSEIDON group 3 (POR patients below the age of 35 years) represent a much easier to treat entity than their older counterparts. In general their chance of producing aneuploid embryos is considerably lower than in women of older age. According to Franasiak et al. (2) the aneuploidy rate identified on the basis of 221 trophectoderm biopsies is 31.3% at the age of 34 years, increasing steadily to over 80% at age 43 and onwards. Therefore, the likelihood of transferring a euploid embryo is high in POSEIDON group 3 patients, even in cases where only two embryos develop from fertilized oocytes. This nevertheless underlines the importance of maximizing the number of good quality mature oocytes by choosing the best individual stimulation approach possible (3). Because there may be considerable high individual variation in the rate of oocyte aneuploidy and resulting embryos even in young patients as has been shown by Minasi et al. (4) it may be worth to clarify the situation of chromosomal problems in the oocytes at an early stage of treatment—i.e., during the first treatment cycle. In countries where embryo biopsy is legally not permitted (like Germany) this can be achieved by performing biopsies on the two polar bodies from normally fertilized oocytes. This will cover only the maternal contribution to chromosomal mal-distribution which nevertheless represents the vast majority of these problems. The high concordance rate of polar body results and the chromosomal constitution of the corresponding oocytes has been well-documented (5). If the results for an individual patient show normal-for-age aneuploidy rates subsequent therapies can focus on optimization of oocyte yield while PGT-A may be added as an adjunct technology for cases identified to have higher rates to spare the patient unnecessary transfers or spontaneous abortions. This may facilitate even POR patients of younger age to shorten the time-to-pregnancy or rather time-to-Live-Birth.

The paper by Haahr et al. (1) is presently the best available guidance for the clinician faced with patients presenting with reduced ovarian reserve to individually tailor the approach to therapy to offer the maximum chance for pregnancy and birth. The additional detailed presentation of information on adjuvant therapies opens the path for further clinical research about their relevance in improving the perspective for all POR patients. Especially for women meeting the POSEIDON group 3 criteria this is the perfect assistance to enable the achievement of live birth rates above 20% by taking the best possible path from the very beginning of the treatment.

## Author Contributions

VB prepared the manuscript. RF reviewed the final version.

### Conflict of Interest

The authors declare that the research was conducted in the absence of any commercial or financial relationships that could be construed as a potential conflict of interest.
